# The Role of Astrocytes in the Molecular Pathophysiology of Schizophrenia: Between Neurodevelopment and Neurodegeneration

**DOI:** 10.3390/biom15050615

**Published:** 2025-04-23

**Authors:** Licia Vellucci, Benedetta Mazza, Annarita Barone, Anita Nasti, Giuseppe De Simone, Felice Iasevoli, Andrea de Bartolomeis

**Affiliations:** 1 Section of Psychiatry, Laboratory of Translational and Molecular Psychiatry, Unit of Treatment-Resistant Psychosis, Department of Neuroscience, Reproductive Sciences and Dentistry, University Medical School of Naples “Federico II”, Via Pansini 5, 80131 Naples, Italy; 2 Department of Translational Medical Sciences, University of Naples “Federico II”, Via S. Pansini 5, 80131 Naples, Italy; 3 Departament de Medicina, Facultat de Medicina i Ciències de la Salut, Institut de Neurociències, Universitat de Barcelona (UB), c. Casanova, 143, 08036 Barcelona, Spain; 4 Institut d’Investigacions Biomèdiques August Pi i Sunyer (IDIBAPS), c. Villarroel, 170, 08036 Barcelona, Spain; 5 Bipolar and Depressive Disorders Unit, Hospìtal Clinic de Barcelona. c. Villarroel, 170, 08036 Barcelona, Spain

**Keywords:** astrocytes, schizophrenia, neurodevelopment, neurodegeneration, antipsychotics, postsynaptic density, treatment resistant schizophrenia, xanomeline, brexpiprazole, clozapine

## Abstract

Schizophrenia is a chronic and severe psychiatric disorder affecting approximately 1% of the global population, characterized by disrupted synaptic plasticity and brain connectivity. While substantial evidence supports its classification as a neurodevelopmental disorder, non-canonical neurodegenerative features have also been reported, with increasing attention given to astrocytic dysfunction. Overall, in this study, we explore the role of astrocytes as a structural and functional link between neurodevelopment and neurodegeneration in schizophrenia. Specifically, we examine how astrocytes contribute to forming an aberrant substrate during early neurodevelopment, potentially predisposing individuals to later neurodegeneration. Astrocytes regulate neurotransmitter homeostasis and synaptic plasticity, influencing early vulnerability and disease progression through their involvement in Ca^2^⁺ signaling and dopamine–glutamate interaction—key pathways implicated in schizophrenia pathophysiology. Astrocytes differentiate via nuclear factor I-A, Sox9, and Notch pathways, occurring within a neuronal environment that may already be compromised in the early stages due to the genetic factors associated with the ‘two-hits’ model of schizophrenia. As a result, astrocytes may contribute to the development of an altered neural matrix, disrupting neuronal signaling, exacerbating the dopamine–glutamate imbalance, and causing excessive synaptic pruning and demyelination. These processes may underlie both the core symptoms of schizophrenia and the increased susceptibility to cognitive decline—clinically resembling neurodegeneration but driven by a distinct, poorly understood molecular substrate. Finally, astrocytes are emerging as potential pharmacological targets for antipsychotics such as clozapine, which may modulate their function by regulating glutamate clearance, redox balance, and synaptic remodeling.

## 1. Introduction

Schizophrenia is a chronic and severe psychiatric disease affecting approximately 1% of the worldwide population. While antipsychotic drugs are the primary treatment, nearly 30% of patients fail to respond to at least two pharmacological trials of antipsychotics from different classes, administered at adequate doses and for a minimum of six weeks each [1]. These individuals are classified as having treatment-resistant schizophrenia (TRS) [2]. Clinical symptoms typically emerge during adolescence or early adulthood and are conceptualized within the framework of an aberrant neurodevelopmental trajectory, characterized by a disruption in synaptic plasticity and cortical–subcortical connectivity [3]. According to the ‘two-hit’ hypothesis, an initial maladaptive event—driven by genetic susceptibility—occurs early in life, altering neurodevelopmental processes. This is followed by a second environmental insult, occurring either early or later in life, which ultimately contributes to the onset of schizophrenia characterized by a complex cluster of symptoms, including hallucinations, delusions, social withdrawal, and impaired executive function [4,5,6,7,8]. Notably, in a rodent model, exposure to a ‘three-hits’ paradigm, rather than two, leads to an exacerbation of psychosis-like behaviors, suggesting the cumulative effect of multiple risk factors on disease severity. This model incorporates genetic susceptibility, early-life stress (such as maternal separation at postnatal days 9–10), and a late environmental insult, exemplified by tetrahydrocannabinol exposure between postnatal days 32-52. Notably, rats subjected to the three-hit model exhibit distinct morphological alterations of the brain compared to those exposed to the two-hit paradigm or controls, including a significant reduction in hippocampal volume [9]. Schizophrenia is characterized by significant functional and cognitive impairments throughout life, historically leading to its classification as dementia praecox. Deficits in working memory and social cognition, alongside evidence of accelerated aging, such as telomere shortening and brain volume loss, have supported the hypothesis that schizophrenia may involve neurodegenerative processes [10,11]. In this context, a unifying theory has been proposed, suggesting that aberrant neurodevelopment processes and subsequent circuit dysfunction may later contribute to neurodegeneration [6]. Specifically, excessive stimulation of the N-methyl-D-aspartate receptor (NMDAR) during development may lead to excitotoxic damage, resulting in the loss of glutamatergic neurons or NMDAR hypofunction [12,13]. This, in turn, may trigger programmed cell death, contributing to the early deficit in executive functions characteristic of schizophrenia [14,15]. Moreover, glutamate hypofunction directly leads to mesocortical hypodopaminergia and mesolimbic hyperdopaminergia—key mechanisms underlying the core symptoms of schizophrenia [16]. Additionally, glutamatergic dysfunction is implicated in increased oxidative stress, potentially driving non-canonical neurodegenerative processes in schizophrenia [6]. This model proposes a unified trajectory of schizophrenia, spanning from early neurodevelopmental disruptions to the onset of full-blown disease, potentially culminating in neurodegeneration. The phenotypic manifestations appear to be linked to windows of vulnerability, with pathological triggers occurring at different stages of neurodevelopment, including embryonic phases or adolescence [6]. This hypothesis underscores the role of neurodevelopmental windows, supported by evidence from linkage analysis and gene expression studies. These studies identify a network of 160 genes implicated in key brain processes, such as progenitor cell proliferation and differentiation, cell migration, synapse formation, axonal connectivity, brain structure patterning, inflammation, and glial cell development, as contributing to the etiology of schizophrenia [17,18,19,20]. While many of these genes are associated with synaptic function, neuronal alterations alone do not fully account for the cognitive and behavioral deficits observed in both animal models and human studies. This suggests the involvement of additional factors in schizophrenia-like phenotypes [21]. In this context, research has increasingly focused on the neuronal microenvironment, particularly on astrocytes, given their role in bidirectional communication with neurons.

Astrocytes constitute 20-40% of all glial cells, with an average diameter of approximately 150 µm. Human astrocytes are nearly three times larger and more symmetrical than their mouse counterparts [22]. In species, these cells are characterized by the high expression of the glial fibrillary acidic protein (GFAP), which increases with age [23]. In humans, astrocytes are classified into four distinct structural and anatomical subtypes: interlaminar, protoplasmic, varicose projections, and fibrous astroglia. Among these, protoplasmic astrocytes—located in cortical layers III and IV—are particularly relevant to the schizophrenia pathophysiology due to their involvement in tripartite synapses and blood–brain barrier (BBB) formation [22,24]. Evidence suggests that the astrocytes-to-neuron ratio has increased throughout evolution, exceeding 1 in the human brain (approximately 1 for the entire brain and 1.4 for the cerebral cortex). While this does not necessarily correlate with greater cognitive capacity, it likely provides the metabolic support required for the heightened energy demands of a larger brain [25,26]. Notably, a single human protoplasmic astrocyte can envelop between 270,000 and 2,000,000 synapses, compared to the significantly lower range of 20,000 to 120,000 synapses in rodents [24]. This suggests that human astrocytes play a crucial role in modulating interneuronal communication by integrating signals from an extraordinarily large number of synapses. Consequently, dysfunction in these astrocytes could profoundly impact neuronal function and network dynamics [22].

**Figure 1:**
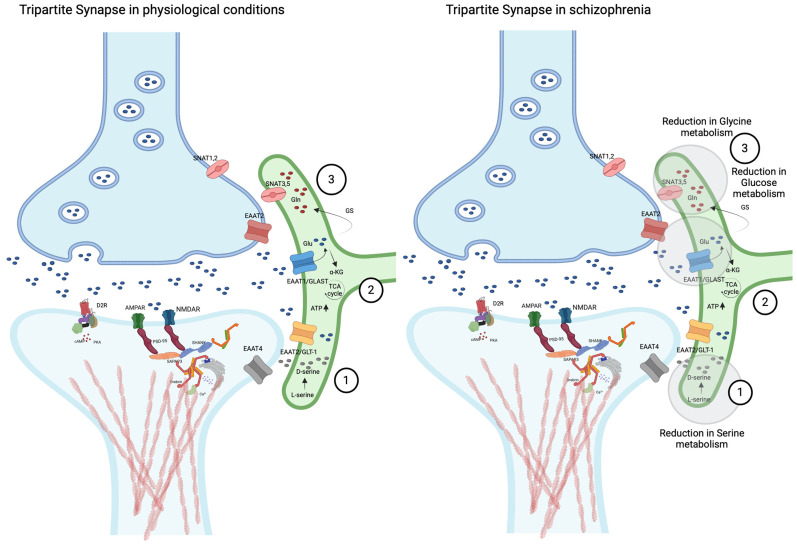
Structure of the tripartite synapse in physiological and pathological conditions typical of schizophrenia with a focus on glutamate homeostasis. Astrocytes play a key role in regulating glutamate neurotransmission through three main mechanisms: (1) via glycine and D-serine metabolism, which modulate NMDAR activity and contribute to synaptic plasticity; (2) via glucose metabolism, which supports energy production through the tricarboxylic acid cycle; and (3) via glutamate clearance and conversion to glutamine, which regulate extracellular glutamate levels and prevent excitotoxicity. In schizophrenia, these regulatory pathways are impaired: the synthesis and availability of D-serine and glycine are reduced, leading to an impairment of NMDAR function, as well as of glucose metabolism, contributing to excitatory/inhibitory imbalance. SNAT, sodium neutral amino acid transporter; EAAT, excitatory amino acid transporters; GLAST, glutamate/aspartate transporters; GLT-1, glutamate transporter 1; Gln, glutamine; α-KG, α-ketoglutaric acid; TCA cycle, tricarboxylic acid cycle; Glu, glutamate; D2R, dopamine receptor 2; PSD-95, postsynaptic density protein 95; NMDAR, N-methyl-D-aspartate receptor; AMPAR, α-amino-3-hydroxy-5-methyl-4-isoxazolepropionic acid receptor; cAMP, cyclic adenosine monophosphate; SAPAP, SAP90/PSD-95-associated protein. Created with Biorender.com.

Astrocytes play a key role in tripartite synapses under both physiological and pathological conditions, particularly in neurotransmitter homeostasis, with a key function in glutamate regulation [27,28] (Figure 1). Additionally, astrocytes express dopamine D1 and D2 receptors (D1R and D2R, respectively) on their membrane surface, positioning them as a critical component of the dopamine–glutamate interaction, a central mechanism in schizophrenia-related neurotransmitter dysfunction.

Emerging evidence further supports the involvement of astrocytes in dopamine regulation within the ventral tegmental area (VTA). In vivo studies have shown that the selective activation of astrocytes enhances the burst firing of dopaminergic neurons, an effect potentially linked to long-term potentiation (LTP) at the excitatory synapses targeting adjacent dopaminergic neurons. This process is dependent on astrocytic Ca^2+^ signaling, mediated via cannabinoid receptor (CB1) and D2R activation within the astrocytic process [29].

Astrocytes play a fundamental role in regulating glucose metabolism, maintaining ion homeostasis maintenance, and balancing pH levels [30,31]. Neuroimaging studies have demonstrated altered cerebral glucose metabolism in patients with schizophrenia, with differences observed based on treatment response [32,33,34,35]. Further supporting the pathological involvement of astrocytes, a recent study identified significant correlations between astrocytic transcriptional profiles in individuals with schizophrenia and those at high risk of psychosis, and the regional pattern of cerebral blood flow phenotypes in both groups [36]. Key predictors of this regional cerebral blood flow phenotype included dopamine-related proteins (D1R, D2R, dopamine transporter—DAT), acetylcholine, γ-aminobutyric acid receptor type A (GABA_A_), and NMDAR [36].

Based on this framework, this comprehensive review aims to address the following questions:

1. Could astrocytes represent a point of convergence between neurodevelopment and neurodegeneration in schizophrenia due to aberrant programming during early development?
2. What are the molecular mechanisms at the tripartite synapse that may influence the schizophrenia pathophysiology?
3. Could astrocytes serve as a potential therapeutic target for schizophrenia?

With these questions in mind, we propose a potential trajectory for astrocyte involvement across different disease stages. Specifically, we explore their role during early neurodevelopment, where their dysfunction may contribute to disease susceptibility, and in later stages, where they may create an unfavorable microenvironment that accelerates premature aging and degenerative processes underlying both prodromal and residual cognitive symptoms.

## 2. Search Strategy for the Comprehensive Review

This review provides a comprehensive and updated synthesis of current evidence on the role of astrocytes in the pathogenesis of schizophrenia, tracing their involvement from neurodevelopmental origins to potential neurodegenerative processes. The EMBASE, Scopus, and Medline/PubMed databases were queried on 10 December 2024. They were last updated on 27th February 2025. Additional articles were manually sourced by reviewing the reference lists of the selected studies and cross-referencing keywords that emerged during the review process. We considered eligible English-language articles published in peer-reviewed journals, exploring the role of astrocytes in schizophrenia during neurodevelopmental and neurodegeneration processes; their involvement in aberrant glutamate, dopamine, and GABA neurotransmission; their role in oxidative stress and synaptic plasticity processes; as well as their role in responses to antipsychotics. No time constraints were applied, and original clinical and preclinical articles were included.

## 3. Astrocyte–Neuron Crosstalk in Neurodevelopmental and Neurodegenerative Processes: Inference for Schizophrenia

### 3.1. The Role of Astrocytes in Schizophrenia: Molecular Basis and Clinical Evidence

Astrocytes, derived from the ectoderm, play a critical role in maintaining central nervous system (CNS) homeostasis. They are involved in neurotransmitter recycling, ionic balance, synaptogenesis modulation, synaptic transmission, and BBB integrity through the neurovascular unit (NVU) [37,38,39,40].

Postmortem studies have revealed significant differences in astrocyte density, morphology, and marker expression between individuals with schizophrenia and healthy controls. Notably, increased astrocyte density has been reported in the hippocampus of schizophrenia patients [41], while reductions have been reported in the prefrontal cortex (PFC), anterior cingulate cortex (ACC), primary motor cortex, and subcortical regions [42,43,44]. At the molecular level, schizophrenia has been associated with the decreased expression of glutamate transporter-1 (GLT-1)—also known as excitatory amino acid transporter 2 (EAAT2) [45]—as well as reductions in GFAP [46], aldehyde dehydrogenase 1 (ALDH1), Ca^2+^-binding protein (S100A4), aquaporin 4 (AQP4), glutaminase, and thrombospondin (TSB1) expression [42,47,48,49].

Genetic studies have further implicated astrocytic dysfunction in schizophrenia. Mutations in the disrupted-in-schizophrenia 1 (DISC1) gene have been identified in the cortical astrocytes of schizophrenia patients [50,51,52]. In preclinical models, microglial DISC-1 mutations have been linked to schizophrenia-like behavioral phenotypes [53,54,55,56,57]. DISC1 knock-out (KO) models exhibit reduced astrocyte spatial density and altered levels of glutamate transporters (GLAST)—also known as excitatory amino acid transporter 1 (EAAT1)—leading to excitatory/inhibitory imbalance [58] (Table 1). Additionally, mutations in astrocytic GLAST have been associated with schizophrenia-like symptoms, some of which are partially reversed by antipsychotic treatment [59,60,61] (Table 1).

Further supporting the role of astrocytes in schizophrenia, a recent study demonstrated that the transplantation of glial progenitor cells (GPCs) derived from schizophrenia patients into neonatal mice resulted in significant alterations in astrocyte differentiation and morphology. These changes, absent in mice receiving GPCs from healthy controls, induced structural and behavioral abnormalities relevant to schizophrenia [62].

**Table 1:** Preclinical models for astrocyte involvement in schizophrenia. DISC1, disrupted-in-schizophrenia 1; KD, knock-down; KO, knock-out; EAAT1/2, excitatory amino acid transporter 1 and 2; GLT-1, glutamate transporter 1; iPSC, induced pluripotent stem cell; SCZ, schizophrenia; MIA, maternal immune activation; IL-6, interleukin 6; IL-1β, interleukin 1 beta; MK-801, dizocilpine maleate; NMDAR, N-methyl-D-aspartate receptor; FABP7, fatty acid binding protein 7, mPFC, medial prefrontal cortex; mEPSCs, spontaneous miniature excitatory postsynaptic currents; GLAST, glutamate transporters; GABA, γ-aminobutyric acid.

Genetic or Pharmacological Animal Model	Species	Main Molecular or Behavioral Findings	Implication for Schizophrenia	References
DISC1 KD	Mice	Reduced astrocyte density and altered levels of GLAST that result in glutamate/GABA imbalance	Excitatory/inhibitory imbalance	[58]
GLT-1 KO	Mice	Increased excitotoxicity and schizophrenia-like behavior	Glutamate dysregulation	[63]
MIA model	Rat	Astrocyte activation with increased IL-6 and IL-1β; long-term changes in synaptic pruning	Neuroinflammation relevant for schizophrenia vulnerability	[64]
EAAT2 blockade, via dihydrokainic acid	Rat	Anhedonia and impaired spatial memory	Dysfunctions that model schizophrenia’s negative and cognitive symptoms	[65]
Inducible expression of mutant DISC1 (ΔC-hDISC1) in astrocytes	Mice	Reduction in D-serine levels in the mice brains	Defects in astrocyte maturation related to NMDAR hypofunction during neurodevelopment	[53]
FABP7 KO	Mice	Reduction in number of synapses in mPFC; reduction in both amplitude and frequency of mEPSCs	Impaired astrocyte lipid signaling affects postnatal cognition; hyperactivity and anxiety-related phenotypes	[66,67]

### 3.2. Astrocytes and Neurodevelopment: Focus on Molecular Mechanism in Schizophrenia

#### 3.2.1. Astrocytes as Regulators of Synaptic Formation and Neurotransmission: Relevance for Schizophrenia Pathophysiology

Schizophrenia is increasingly recognized as a neurodevelopmental disorder arising from the complex interplay of genetic and environmental risk factors during the pre- and postnatal periods. Genetic susceptibility is reflected in the polygenic risk score [68], and environmental influences include obstetric complications, fetal abnormalities, maternal stress during pregnancy, and infectious disease during pregnancy—which may lead to hyperactivation of the hypothalamic–pituitary–adrenal axis [69,70,71,72,73]. The convergence of these environmental stressors with genetic vulnerabilities—such as variations in transcription factor sp4 (SP4), histone–lysine N-methyltransferase SETD1A, triple functional domain protein (TRIO), zinc finger protein 804A (ZNF804A), contactin-4 (CNTN4), leucine-rich repeat-containing protein 4B (LRRC4B), and netrin receptor DCC (DCC) [74,75,76,77]—is thought to contribute to the occurrence of schizophrenia symptoms [78].

Initially proposed by Weinberger [79] and later supported by experimental findings, the neurodevelopmental hypothesis of schizophrenia suggests that disrupted early brain maturation leads to impaired synaptic plasticity and myelination. These changes, such as a loss of hippocampal oligodendrocytes and volume reduction in the neuronal Papez circuit, ultimately impair brain connectivity [80,81,82,83].

Glutamatergic dysfunction, a central mechanism in schizophrenia and a key contributor to dopaminergic dysregulation, may originate from early neurodevelopmental disruptions. Given that sustained glutamatergic activity is critical for neuronal survival, early deficits may contribute to the loss of glutamatergic neurons [6,14]. During adolescence, NMDAR hypofunction may lead to excessive synaptic pruning due to the lack of glutamatergic-driven plasticity [84,85,86]. This disruption in glutamatergic control over dopaminergic pathways, particularly in the PFC and VTA, is thought to precipitate symptom onset in adolescence [16]. Moreover, excessive dopaminergic activity and phasic glutamatergic stimulation—particularly in the absence of effective pharmacological treatment or in cases of treatment resistance—may exacerbate excitotoxic brain damage, potentially through increased oxidative stress [6,87]. Given the central role of glutamate in these processes and the critical involvement of astrocytes in their metabolism, emerging evidence suggests that astrocytes may contribute to the establishment of a vulnerable substrate for neurodegeneration during critical neurodevelopmental periods.

During both neurodevelopment and later stages of life, astrocytes favor synapse formation and maturation, maintaining neurotransmitter homeostasis and supporting neuronal survival [88,89]. Astrocytes are not just silent spectators [89], but they are instead identified as “choreographers” of the neural circuit assembly [89]. Astrocytic signaling enhances neuronal receptivity to synaptogenesis, particularly during brain development and neurorepair through different pathways including that of brain-derived neurotrophic factor (BDNF)–tyrosine protein kinase (TrkB) and that of transforming growth factor-b (TGF-β)–T-box brain transcription factor 1/2 (TBR1/2) [90] (Table 2). In addition to their role in synaptic development, astrocytes are also involved in glutamate homeostasis [91,92]. Glutamate is synthesized from α-ketoglutarate via the astrocyte–neuron glutamate–glutamine cycle, where neuronal glutaminase converts glutamine into glutamate [93]. To prevent excitotoxicity, excess glutamate is rapidly cleared from the synaptic cleft by neuronal and astrocytic EAAT1/2 [94,95]. This clearance mechanism limits the prolonged stimulation of NMDAR and α-amino-3-hydroxy-5-methyl-4-isoxazolepropionic acid receptor (AMPAR) that could induce a Ca^2+^ overload and neuronal death [96]. Additionally, glutamate levels in the synaptic cleft are finely tuned by the cycle of glutamine obtained by the conversion of glutamate, thanks to the astrocyte enzyme glutamine synthetase [97] (Table 2). This mechanism ensures low levels of glutamate in the synapsis, avoiding the risk of excitotoxicity while maintaining neurotransmitter availability.

The critical importance of this regulatory system is highlighted by findings in individuals with schizophrenia who carry single nucleotide polymorphisms in both astrocytic and neuronal EAAT1/2. These genetic variants are associated with reduced transporter expression and activity and correlate with poorer performance at the Wisconsin Card Sorting Test, which evaluates cognitive flexibility [98]. These observations are further confirmed by preclinical studies. EAAT1-deficient mice (EAAT1^−/−^) display significant impairments in learning and memory, particularly in social recognition tasks [60]. Similarly, pharmacological EAAT2 blockade, using dihydrokainic acid, leads to deficits in spatial learning and short-term memory deficits in rat models [65] (Table 1).

In conclusion, abnormal glutamate transmission at the tripartite synapse is responsible for aberrant astrocyte–neuron signaling that results in abnormal cell development, which impacts circuit formation and function, putatively hesitating in cognitive dysfunctions, as reported in animal models [60,99,100].

**Table 2:** Main astrocyte molecular mechanisms which are potentially involved in schizophrenia. BDNF, brain-derived neurotrophic factor; TrkB, tropomyosin receptor kinase B; TGF-β, transforming growth factor beta; TBR1/2, T-box brain transcription factors 1 and 2; NLG, neuroligin; FABP7, fatty acid binding protein 7; AQP4, aquaporin 4; claudin-5, tight junction protein claudin-5; ICAM-1, intercellular adhesion molecule 1; Shh, sonic hedgehog; EAAT1/2, excitatory amino acid transporter 1 and 2; glutamine synthetase, enzyme for glutamate detoxification and recycling; NMDAR, N-methyl-D-aspartate receptor; A2aR, adenosine A2a receptor; D2R, dopamine D2 receptor; VMAT2, vesicular monoamine transporter 2; OCT3, organic cation transporter 3; Kir1.4, inward rectifier potassium channel; Na⁺/K⁺ ATPase, sodium/potassium-transporting ATPase; IL-1β, interleukin 1 beta; TNF-α, tumor necrosis factor alpha; NF-κB, nuclear factor kappa B; NLRP3, NOD-, LRR-, and pyrin domain-containing protein 3; COX-2, cyclooxygenase-2; ROS, reactive oxygen species; STAT3, signal transducer and activator of transcription 3.

Domain	Astrocyte Role	Molecular Mechanisms	Functional Effect	References
Neurodevelopment	Regulation of synaptogenesis and dendritic development	BDNF/TrkB—TGF-β/TBR1/2	Synaptogenesis	[66,90,101,102]
		NLG	Synaptic pruning	
		FABP7	Dendritic maturation	
	Control of BBB development and integrity	AQP4	BBB development	[103,104,105]
		Claudin-5 and ICAM-1 via Shh pathway	BBB integrity	
Neurotransmission	Glutamate	EAAT1/2	Extracellular glutamate level regulation and prevention of excitotoxicity	[94,95,96]
		NMDAR		
		Glutamine synthetase		
	GABA	A2aR activation through the gephyrin pathway	Improvement of GABAergic stability	[106]
	Dopamine	Ca^2^⁺ signaling	Dopamine vesicle transport and reuptake in glial cells	[107,108]
		VMAT2 and OCT3		
Neurodegeneration	Metabolic and ionic homeostasis	Kir1.4 and Na⁺/K⁺ ATPase	Stabilization in ionic gradients	[109,110]
	Oxidative stress and neuroinflammation	IL-1β, TNF-α, NF-κB, NLRP3, COX-2	Driving inflammation	[111,112,113]
		ROS and STAT3	Stress response regulation	

#### 3.2.2. Disruptions in Astrocytic Function and Implications for Schizophrenia Pathophysiology

The neuroligin family (NLGs), although well characterized in neurons, remains poorly understood in terms of its function in astrocytes. These molecules have been strongly implicated in multiple neurodevelopmental and neuropsychiatric conditions, including autism spectrum disorder (ASD) and schizophrenia. While neuroligins are known to mediate trans-synaptic signaling and contribute to the shaping of neural circuits, their specific role through astrocyte-mediated mechanisms is still under investigation [114]. A recent study explored the effects of astrocyte-specific deletion of NLG3 in the ventral hippocampus of adult male mice. At the behavioral level, this manipulation resulted in impairments in social memory, which represent a hallmark of many neurodevelopmental and neuropsychiatric disorders [102]. These behavioral abnormalities were accompanied by deficits in LTP, potentially linked to altered Ca^2+^ signaling and the upregulated expression of EAAT2 [102,115,116,117].

Astrocytes regulate neurogenesis and glutamatergic transmission through the synthesis of D-serine, a co-agonist of NMDAR, which exerts a significant modulatory effect on glutamate receptor activity [118]. D-serine is derived from L-serine by serine racemase action located predominantly in the astrocytes [119]. In a mouse model with the inducible expression of the human mutant DISC1 gene, the mutant DISC1 protein fails to interact with serine racemase. This disrupts enzyme stability by promoting its ubiquitination and degradation, leading to a marked reduction in D-serine production. As a result, these mice exhibit increased sensitivity to NMDAR antagonists such as MK-801 (an effect counteracted by D-serine administration) [53] (Table 1). This preclinical model also displays the reduced proliferation of neural progenitors, the abnormal maturation of hippocampal neurons, and behavioral deficits reflective of emotional and cognitive dysfunctions. These abnormalities are enhanced by D-serine administration, which normalizes the neuron’s development and improves behavioral impairments [54]. These results suggest that mature astrocytes contribute to hippocampus-dependent affective and cognitive functions by regulating adult hippocampal neurogenesis and participating in key neurodevelopmental processes [54,120].

#### 3.2.3. Astrocyte–Neuron Interactions: The Tripartite Synapse

A transcriptional study using induced pluripotent stem cell (iPSC)-derived neurons from 80 donors demonstrated that astrocyte–neuron interactions significantly enhanced synaptic gene expression in neurons. This interaction also led to the increased expression of synaptic adhesion molecules in astrocytes, making it one of the best-supported iPSC study models of the astrocyte/neuron interplay [121].

Notably, gene expression profiles from both cell types were enriched for schizophrenia-associated risk genes, highlighting the pivotal role of astrocytes in conferring susceptibility to the disorder [121]. In animal models of neurodevelopmental disorders, including Rett, Fragile X, and Down syndromes, an upregulation of both astrocyte bone morphogenetic protein (BMP) and insulin-like growth factor binding protein 2 was observed. While these molecules promote astrocyte differentiation under physiological conditions, their overexpression in pathological states contributes to aberrant neuronal development [122]. Moreover, GPCs isolated from individuals with early-onset schizophrenia exhibited impaired astrocytic differentiation. This defect was rescued by inhibiting bone morphogenetic protein (BMP) signaling through the knock-down of mothers against decapentaplegic homolog 4 (SMAD4), further implicating dysregulated astrocytic development in the pathophysiology of schizophrenia.

Astrocytic Ca^2+^ dysregulation was found to be implicated in an animal model of neurodevelopmental disorders [123,124]. Wang and colleagues demonstrated that intracellular Ca^2+^ signaling mediated by inositol 1,4,5-trisphosphate receptor (IP_3_R) type 2 in astrocytes can drive developmental impairments [124]. Astrocytic Ca^2^⁺ signaling critically regulates neuron–glia interactions, influencing neurotransmitter release and overall network dynamics [125]. This signaling process can be triggered by several neurotransmitters, including glutamate, dopamine, adenosine triphosphate (ATP), adenosine diphosphate (ADP), histamine, or acetylcholine [126,127]. The application of glutamate to mature human astrocyte cultures induces a dose-dependent increase in intracellular Ca^2^⁺ levels via the activation of metabotropic glutamate receptor 5 (mGluR5) [128]. However, this response is observed only in astrocytes derived from pups after postnatal week 3 [129]. Moreover, mGluR5 expression in mature human astrocytes was low, suggesting that this mechanism may be related to a particular developmental stage, with alternative pathways mediating Ca^2^⁺ signaling in adulthood [128]. Interestingly, Ca^2+^ responses in astrocytes also appear to be heterogeneous in mice, varying based on the specific neurotransmitter involved—further emphasizing the complexity and context-dependence of astrocytic signaling. It has been hypothesized that the heterogeneity of astrocytic Ca^2+^ signaling may be linked to distinct temporal kinetics, thereby refining the specificity of the transmitted signal. This temporal precision could allow astrocytes to selectively encode incoming stimuli and respond appropriately to neuronal input [22]. Additionally, subcellular compartments—including the soma, branches, and processes—may support localized Ca^2+^ signal patterns that operate with a degree of independence [22,130,131,132]. The nature of astrocytic Ca^2+^ dynamics is also shaped by the type of astrocyte–neuron interaction, which occur at three levels: (i) at individual synapses, where Ca^2+^ transients within a single tripartite synapse influence neuronal excitability, synaptic plasticity, and neurotransmitter release; (ii) across groups of synapses, where astrocytic Ca^2+^ signaling affects the plasticity and activity of multiple synaptic ensembles; and (iii) at the network level, where Ca^2+^ waves propagate through astrocytic networks, thereby coordinating neuronal network activity and information processing [125]. The activation of metabotropic receptors on astrocytes by neurotransmitters like glutamate, dopamine, and noradrenaline leads to an increase in intracellular Ca^2+^ concentration, generating spike-like Ca^2+^ responses [133,134,135,136,137,138,139,140]. The canonical mechanism underlying astrocytic Ca^2+^ responses involves the release of Ca^2+^ from intracellular stores, primarily from the mitochondria and the endoplasmic reticulum (ER) [137,141]. ER-mediated Ca^2+^ release is facilitated through three distinct types of IP_3_R channels [137,141,142]. Additionally, other mechanisms contribute to Ca^2^⁺ homeostasis, including membrane transporters such as the EAAT1/2, the Na^+^/K^+^-ATPase, and the Ca^2+^/Na^+^ exchanger (NCX) [137,143,144]. The preferential mechanism of Ca^2^⁺ regulation depends on the astrocyte’s surface/volume ratio: in regions with a high surface/volume ratio, the predominant mechanism is ER-mediated release, whereas in regions with a lower ratio, membrane transporters and exchangers play a more significant role [143]. The disruption of these processes—potentially due to early-life exposure to abnormal glutamate signaling—may contribute to aberrant circuit formation, ultimately leading to dysfunctional neuronal networks and associated clinical phenotypes. These findings are confirmed by a recent preclinical study in which the transplantation of ASD-derived astrocytes into healthy mouse brains is found to be responsible for cognitive-like and behavioral deficits [123].

Astrocytes also regulate synaptic activity through adenosine neurotransmission, which is closely linked to intracellular Ca^2+^ signaling. Astrocyte-derived ATP plays a key role in behavior modulation, primarily through P2X2 receptors [124]. Elevated intracellular Ca^2+^ levels trigger ATP release from astrocytes, which is rapidly converted into adenosine. At excitatory glutamatergic synapses, adenosine primarily acts through the adenosine A1 receptor (A1R) to inhibit glutamate release [145,146]. Conversely, at inhibitory synapses in the postnatal hippocampus—particularly during synaptogenesis—adenosine modulates GABAergic neurotransmission via the adenosine A2a receptor (A2aR), whose activation stabilizes synapses via the gephyrin pathway [106]. When astrocytic Ca^2+^ dynamics are disrupted, adenosine signaling becomes dysregulated. This can impair the stabilization of both pre- and postsynaptic elements. Critically, if A2aR remains inactive for over 20 min, it can cause irreversible synapse destabilization and synapse loss, events that may underlie the cognitive deficits commonly observed in schizophrenia [106].

#### 3.2.4. Environmental and Genetic Factors Influencing Astrocyte Function

Neurons are protected by the BBB, which provides a tightly regulated environment essential for proper neurodevelopment [147,148,149], reinforcing the brain’s “immune privilege” status [150]. The integrity and function of the BBB are maintained through complex signaling pathways, including astrocytic sonic hedgehog signaling (Shh). This pathway promotes the expression of key tight junction proteins such as claudin-3, claudin-5, occludin, and cell adhesion molecule-A [103] (Table 2). However, Shh signaling varies across brain regions and is influenced by astrocyte subtypes and their responses to environmental and inflammatory stimuli [151,152]. In schizophrenia, reduced astrocyte density has been documented in several brain regions of schizophrenia patients, including the subgenual cingulate, anterior, dorsolateral, prefrontal cortices, hippocampus, and corpus callosum [153]. This reduction contributes to abnormal BBB architecture, notably through the decreased expression of AQP4, which is relevant to BBB development, integrity, and intracerebral water homeostasis [104]. On the other hand, a decrease in claudin-5 protein, among the other twelve tight junction proteins, has been observed in schizophrenia brains compared to healthy controls [154] and is correlated with the age of onset and duration of disease [155]. The role of the BBB in schizophrenia becomes even more complex considering the existence of the NVU, constituted by endothelial cells, pericytes, and astrocytes, which regulate both the cerebral blood flow and neuronal microenvironment [156,157,158]. Disruption in NVU function can lead to imbalanced levels of neurotransmitter (e.g., glutamate and dopamine) levels, with subsequent disrupted cellular signaling and neuron damage, which result in the cognitive and behavioral manifestations of schizophrenia [159]. Structural abnormalities in NVU components have been observed in the postmortem brains of schizophrenia patients. These include a reduced number of GFAP-positive astrocytes surrounding blood vessels in the PFC, hippocampus, and ACC [160,161]. Furthermore, reductions in capillary density, endothelial cell degeneration, and disruptions in astrocytic endfeet and basement membranes have been reported in the PFC and visual cortex. These changes are associated with negative symptoms and may influence responsiveness to antipsychotic treatment [150].

Disruptions in BBB integrity during early development can expose the brain to harmful environmental agents, including infectious stimuli, chemicals, and other toxic compounds, which are strongly associated with aberrant neurodevelopment. Among these, endocrine disruptors that cross the BBB, such as bisphenol A (BPA), phthalates, and polychlorinated biphenyls, can cross the BBB and interfere with sex hormone signaling, a critical process for brain development. Experimental models have used endocrine disruptor exposure to stimulate neurodevelopmental disorders, demonstrating that these compounds can trigger acute and chronic neuroinflammatory responses mediated by astrocytes and microglia [162,163], suppress the proliferation of cortical pyramidal neurons [164], and disrupt neuronal migration—ultimately contributing to the formation of dysfunctional neuronal circuits, a pathological feature observed in schizophrenia [165]. A recent study by Al-Shami and colleagues examined the molecular effects of BPA exposure during critical postnatal periods in rats, focusing on markers of autophagy and neuroinflammation [64] (Table 1). BPA exposure was found to elevate the expression of pro-inflammatory markers in the PFC and hippocampus, including nuclear factor kappa-light-chain-enhancer of activated B cells (NF-κB), recombinant IL-1 beta equine produced in E.coli cells (eIL-1β), recombinant IL-2 beta equine produced in E. coli cells (eIL-2), and cyclooxigenase-2 (COX-2)—all of which are commonly secreted by activated astrocytes and contribute to cognitive impairments. Interleukin-1β (IL-1β) is especially notable for its role in synaptic dysfunction and neuroinflammation in Alzheimer’s disease, while COX-2 facilitates the production of pro-inflammatory prostaglandins, creating an environment conducive to neurodegeneration. In addition to inflammatory responses, BPA exposure also upregulated autophagic markers such as NOD-, LRR-, and pyrin domain-containing protein 3 (NLRP3), a central component of the inflammasome, and Beclin-1 [64] (Table 2). These molecules are essential for the formation and maturation of autophagosomes, which are involved in cellular degradation and recycling processes. Dysregulation of autophagy disrupts cellular homeostasis and has been increasingly implicated in the pathogenesis of neurodegenerative diseases. A histopathological assay further revealed neuronal loss, particularly among pyramidal and granular cells, two cellular populations critical for hippocampal function and memory processing. Additionally, the findings included neuronophagia, vascular dilatation suggesting BBB compromise, and the presence of flame-shaped microglia, indicative of microglia activation [166,167,168,169].

In summary, astrocytes play a fundamental role in brain development, participating in processes ranging from synapse formation to neurotransmitter regulation and neuronal support. Impaired astrocyte maturation or function may significantly contribute to the pathophysiology of schizophrenia. In this context, the PSD not only underpins proper synaptic functioning but also emerges as a potential target for genetic alterations linked to neurodevelopmental disorders. Further research studies into astrocytic biology are critical for understanding their function in normal brain development and neurodevelopmental disorders like schizophrenia.

### 3.3. The Role of Astrocytes in Neurodegeneration: The Focus on Molecular Mechanisms in Schizophrenia

Schizophrenia was originally described as “dementia praecox” by Kraepelin, despite the lack of canonical histopathological features of neurodegeneration (as instead reported in Alzheimer’s and Parkinson’s diseases). Although the clinical presentation appeared progressive, this notion was later challenged by evidence of a stable neuropsychological deficit detectable from illness onset. This discrepancy suggested that schizophrenia may have involved non-canonical neurodegenerative mechanisms, distinct from classical neurodegeneration [11]. Under physiological conditions, astrocytes are critical regulators of brain homeostasis. These functions include the following: (i) facilitating glucose uptake through glucose transporter 1 (GLUT 1) [170]; (ii) regulating lipid packaging via apolipoprotein E (APOE), which is relevant to maintaining the BBB structure via the suppression of cyclophilin A (CYPA), NF-κB, and metalloproteinase 9 (MMP9) signaling in pericytes, thereby counteracting APOE4-associated BBB breakdown [171,172]; (iii) maintaining extracellular potassium levels through Kir1.4 channels, which are crucial for modulating neuronal membrane potential and excitability [109,173,174]; and (iv) regulating brain water homeostasis via AQP4 regulation. Notably, certain AQP4 single nucleotide polymorphisms (e.g., rs1058424, rs335929, rs3763043) are associated with more severe negative symptoms in schizophrenia and the need for higher doses of olanzapine for symptom control [175,176,177]. Disruption of these astrocytic functions contributes to pathological neurodegeneration. Ca^2+^ deregulation, often due to altered NCX activity, can impair neuronal function, increase the excitotoxicity risk via NMDAR hyperactivation, and disrupt energy metabolism, neuronal excitability, and lipid availability. Moreover, disturbances in intracellular Ca^2+^ signaling, extracellular K^+^ concentrations, and glutamate uptake impair lactate release by astrocytes, an essential energy substrate for neurons [178,179].

Molecular dysfunction in astrocytes frequently leads to astrogliosis, characterized by an abnormal proliferation of astrocytes, along with synaptic loss and the upregulation of genes associated with a pro-inflammatory state. These changes contribute to neuronal damage, particularly within the hippocampal and prefrontal regions, which are critically involved in cognitive processes and frequently implicated in the schizophrenia pathology [180]. Additionally, dysfunctions of astrocytic mitochondrial glutamate dehydrogenase (Glud1) have been linked to elevated glutamate levels in the medial PFC and hippocampus. In animal models, the homozygous deletion of Glud1 has resulted in impairments in working memory, spatial orientation, and social behavior, underscoring the importance of astrocyte-mediated glutamate metabolism in cognitive function [181,182,183,184].

Astrocytes also regulate neuroinflammatory processes thanks to the different cellular subtypes represented by A2 astrocytes (the neuroprotective type), which may produce neuroprotective factors; and the A1 subtype that can adopt neurotoxic profiles, releasing inflammatory cytokines and causing neuronal damage [185,186]. The A1 subtype activates the pro-inflammatory NF-κβ pathway leading to the upregulated expression of complement proteins, including complement component 1 (C1)r, C1s, complement component 3 (C3), and complement component 4 (C4). This activation is often triggered by cytokines and complement proteins secreted by activated microglia, including TNF-α, IL-1α, and C1q [187,188,189]. A1 astrocytes also display a diminished capacity for phagocytosing synaptic and neuronal debris and show impaired support for neuronal growth and function [189]. On the other hand, the transcriptomic profiling of the A2 astrocytic subtype reveals the upregulated expression of both anti-inflammatory and neurotrophic factor genes, such as cardiotrophin-like cytokine factor 1, S100A10, pentraxin 3, sphingosine, kinase 1, IL-6, leukemia inhibitory factor (LIF), transglutaminase 1, Arginase-1, and nuclear factor erythroid 2-related factor 2 [189,190]. Furthermore, genetic mutations can disrupt the release of neuroactive compounds, such as glutamate, leading to excitotoxicity and abnormal spontaneous Ca^2+^ oscillation. These disruptions can trigger reactive astrogliosis—a phenomenon characterized by morphological and functional changes in astrocytes—which in turn is responsible for a cascade of inflammatory responses [185].

In summary, astrocytes influence both glutamatergic neurotransmission and neuroinflammation through their polarization into the neuroprotective A2 or neurotoxic A1 subtype. Genetic mutations that promote reactive astrogliosis may contribute to sustained inflammatory states and neurotoxicity. Both clinical and preclinical findings highlight the pivotal role of astrocytes in neurodevelopmental disruptions and non-canonical neurodegeneration, supporting their potential as therapeutic targets in schizophrenia.

#### Astrocyte–Microglia Crosstalk in Schizophrenia: Relevance for Neuroinflammation in Neurodegeneration Processes

During neurodevelopment, inflammatory stimuli such as interferon-γ (IFN-γ) or lipopolysaccharide (LPS) can trigger the activation of microglia into a reactive state. This inflammatory environment disrupts the differentiation of GPCs, leading to the production of both immature astrocytes and oligodendrocytes. As a result, critical functions such as glutamate and K^+^ buffering, growth factor secretion, and proper myelination are compromised. These alterations contribute to white matter abnormalities and hypomyelination, which may underlie the emergence of schizophrenia symptoms during adolescence, particularly when combined with a later environmental or physiological ‘final hit’ [4,8,9,191,192,193,194,195].

Throughout both early and later life, glial cells engage in bidirectional communication that modulates neurogenesis, BBB integrity, and synaptic development. Astrocytes help maintain microglia in a resting state through signaling molecules such as transforming growth factor β-1 (TGF-β) and ATP. In turn, activated microglia can induce astrogliosis by releasing pro-inflammatory cytokines, including IL-1β and tumor necrosis factor α (TNF-α) [196]. In this context, compounds like cannabidiol and nervonic acid have shown potential in modulating glial activation, restoring glial homeostasis, and alleviating schizophrenia-related neuroinflammation [197,198].

Glial cells also play a role in synaptic pruning. Microglia mediate synapse elimination via complement proteins including C1q and C3, while astrocytes facilitate this process by releasing IL-33. Supporting this mechanism, Purves-Tyson and colleagues reported the upregulation of C1qA, C3, and C4 in the midbrains of patients with schizophrenia [199]. Postmortem studies further corroborate the neuroinflammatory hypothesis, revealing increased microglial activation in affected brain regions [200,201].

Developmental models suggest that both astrocytes and microglia exhibit a shared pro-inflammatory response to postnatal inflammatory stimuli, marked by elevated inducible nitric oxide synthase (iNOS) expression [202]. Preclinical studies have also identified overlapping gene expression profiles in reactive astrocytes and disease-associated microglia, particularly involving NF-κB and toll-like receptor pathways. Additionally, genetic and epigenetic analyses have identified glial dysregulation in schizophrenia risk, including variants (e.g., gamma-aminobutyric acid A receptor beta 2—GABRB2) and demonstrated disruption of astrocyte–microglia crosstalk in KO mouse models [203,204].

Collectively, these findings support the hypothesis that dysregulated astrocyte–microglia interactions may play a significant role in the pathogenesis and progression of schizophrenia.

### 3.4. From the Tripartite to Tetrapartite Synapse Conceptualization: Insights into the Neurobiology of Schizophrenia

#### 3.4.1. Postsynaptic Density Structure and Schizophrenia

The postsynaptic density (PSD) is a highly specialized structure located beneath the glutamatergic postsynaptic membrane [205]. Functioning as a molecular hub, it integrates multimodal intracellular signals from different receptors (e.g., NMDAR, AMPAR, mGluRs, dopamine receptors) by coordinating both the spatial and temporal aspects of signal transduction. The PSD is evolutionarily conserved, containing approximately 1000 shared proteins across vertebrates, with additional proteins contributing to species-specific features [206]. Structurally, the PSD is organized into two distinct layers with a morpho-functional hierarchy: the core, located near the neuronal membrane; and the pallium, an outer layer containing various local proteins such as scaffolding proteins, adaptors, and inhibitory molecules [207]. This architecture acts as a functional and structural hub where multiple neurotransmitters, such as glutamate, dopamine, serotonin, GABA, and glycine, converge, representing a site of the integration of neurotransmitter information essential for the regulation of intracellular signaling pathways.

Recent evidence underscores PSD as a synaptic component essential for the development of dendritic spines and the regulation of multiple intracellular signaling pathways. The dysregulation of these processes is linked to abnormal synaptic plasticity mechanisms (like impaired dendritic spine maturation and reduced LTP) associated with psychiatric disorders, including neurodevelopmental disorders and, particularly, schizophrenia [208,209,210]. Notably, the evidence of PSD’s role in the neurobiology underpinning psychotic disorders is supported by studies showing that antipsychotic treatment significantly affects the gene expression of PSD proteins, including Homer, postsynaptic density protein 95 (PSD-95), Shank, stargazing, and DISC1 [211,212,213,214,215,216,217]. Evidence also suggests that changes in the PSD components directly impact the structure and function of the glial cells. For instance, Homer1 has been implicated in modulating astrocyte phenotypes, promoting a shift from the neurotoxic and pro-inflammatory A1 state to the neuroprotective and anti-inflammatory A2 phenotype. The latter is associated with the release of anti-inflammatory cytokines and neurotrophic factors, contributing to brain homeostasis [218].

Taken together, these data support the hypothesis of direct communication between glutamate PSD and its surrounding microenvironment. This interface plays a crucial role in neuronal maturation and may represent a key target in understanding and treating synaptic dysfunctions in schizophrenia and related disorders.

#### 3.4.2. Regulation of Synaptic Plasticity: Pre- and Postsynaptic Terminals, Glial Cells, and Extracellular Matrix

Brain cells are involved in reshaping brain structures throughout life by generating or eliminating synapses in response to specific stimuli [219,220]. These dynamic processes are finely regulated, and their disruption is implicated in the occurrence of neurodevelopmental abnormalities that underlie the pathophysiology of schizophrenia [221,222].

Within this framework, a key role is played by astrocytes, with their processes enveloping pre- and postsynaptic elements and closely approaching the synaptic cleft. In response to presynaptic activation, astrocytes release ‘gliotransmitters’ such as glutamate, ATP, and GABA [130,223,224,225,226,227,228], which contribute to synaptic modulation and neuronal communication. All these cellular elements are structurally and functionally supported by the extracellular matrix (ECM), which plays a role in synaptic plasticity, forming a complex network that occupies about 20% of the adult brain’s volume, structuring the tetrapartite synapse [229]. Alterations in ECM components, whether driven by genetic mutations or pharmacological interventions, can impair astrocytic Ca^2+^ signaling and synaptic plasticity. For instance, changes in ECM proteins such as tenascin-C and thrombospondins 1 and 2 have been associated with reduced LTP in rodent models [230,231]. Conversely, astrocytes can actively influence ECM composition. Pantozopoulos and colleagues demonstrated that astrocytes could induce the expression of chondroitin sulfate proteoglycans in the lateral nucleus of the amygdala and the lateral entorhinal cortex, contributing to region-specific ECM imbalances that may have affected neurodevelopmental trajectories and synaptic circuit function. The imbalances may have been associated with the impaired secretion or increased internalization of these components by glial cells, potentially contributing to the neurotransmitter imbalances observed in schizophrenia [232,233]. Under physiological conditions, ECM is essential for synapse formation and stabilization during brain development [234]. The altered synthesis of ECM components, including cell adhesion molecule L1 (CHL1), which is involved in regulating both neuronal survival and growth and dendritic spine pruning in developing pyramidal neurons, has been reported both in preclinical and clinical settings [235]. Indeed, abnormal synapse formation and elimination are now widely recognized as central pathological mechanisms in schizophrenia and related neurodevelopmental disorders [236,237,238,239]. Astrocytes play a direct role in the maturation of the brain after birth through multiple mechanisms, like the release of synaptogenic factors, such as fatty acid binding protein 7, which influence both the formation and elimination of synapses (Table 1) [66,67,88,240]. Synaptic pruning is particularly dependent on astrocyte-mediated phagocytosis, a mechanism regulated by Ca^2+^ signaling through IP_3_, leading to Ca^2+^-dependent synaptic elimination [241,242,243,244]. This function also involves the activation of specific phagocytic pathways, including proteins such as MEGF10 (an orthologue of Drosophila Draper [245,246] and Caenorhabditis elegans CED-1) and the MER/AXL/TYRO3 receptor kinase family (MERTK) [241].

#### 3.4.3. Astrocytes’ Role in Dopamine–Glutamate Interplay: Relevance for Schizophrenia

According to the glutamate hypothesis, schizophrenia is associated with circuit-level dysfunctions driven by NMDAR hypofunction, particularly in the PFC of schizophrenia patients. This receptor dysfunction primarily affects the inhibitory control exerted by parvalbumin-positive (PV^+^) GABAergic interneurons, resulting in the disinhibition of glutamatergic pyramidal cells and a subsequent imbalance between excitatory and inhibitory signaling. NMDAR-mediated neurotransmission requires endogenous co-agonists like glycine or D-serine—primarily released by astrocytes [247,248,249,250,251]. During early development, D-serine levels increase in the mammalian brain, reflecting their role in brain maturation via transforming growth factor beta 1 (TGF-β1) [252,253]. People carrying mutations in D-serine racemase [254] and D-amino acid oxidase [255], as well as other enzymes involved in D-serine metabolism [256], are much more likely to have schizophrenia.

Dopamine is the other player in the pathophysiology of schizophrenia, as also demonstrated by the mechanism of action of antipsychotic drugs that, despite differences in receptor profiles, act mainly on the positive symptoms, as antagonists of D2R [42,257,258,259,260,261,262]. The role of astrocytes in the context of dopaminergic activity is confirmed by changes in astrocytic enzymes involved in dopamine metabolism and storage, including enzymes of mitochondria, monoamine oxidase B (MAO-B), and catechol-O-methyltransferase (COMT) [263,264], as well as the plasma membrane organic cation transporter 3 (OCT3) [107,128,265,266] and vesicular monoamine transporter 2 (VMAT2) [128,263] (Table 2). VMAT2 mediates synaptic plasticity within the early developmental stage, as revealed by the elevation of its expression in PFC around the second week after birth in rodents and humans [107,267]. Notably, the conditional deletion of VMAT2, specifically in astrocytes within the PFC of schizophrenia patients, leads to a disruption in dopaminergic homeostasis. This results in excessive tonic dopamine release, further exacerbating deficits in executive functions associated with schizophrenia [107,268].

Taken together, this evidence supports a constant crosstalk between dopamine and glutamate, which also occurs at astrocytic levels. A recent computational study supports this viewpoint showing that astrocytes’ intracellular Ca^2+^ levels are finely modulated by dopamine–glutamate interplay, suggesting that the released dopamine activates astrocytes via dopaminergic receptors (probably via D1R receptors and the IP_3_ pathway), enhancing the initiation and propagation of glutamate-evoked Ca^2+^ signals in a manner dependent on both astrocytic morphology and the spatial distribution of glutamatergic inputs [269].

In summary, these findings suggest that astrocytes are not only involved in maintaining glutamate balance but also in modulating dopamine transmission, especially in the PFC. The expression of VMAT2 in the astrocytes located in the frontal cortex may indicate a targeted function in regulating dopaminergic activity. This regulatory role has significant implications for synaptic plasticity and cognitive functions, both of which are intricately linked to glutamate transmission. Together, the evidence supports the view that astrocytes act as a critical interface between the dopamine and glutamate systems, shaping neural circuit function in healthy individuals and those with the disease.

### 3.5. Aberrant Astrocyte Programming in Neurodevelopment: Implications for Schizophrenia

Astrocytes’ molecular heterogeneity likely originates early in life from distinct developmental trajectories. During a primary neurodevelopmental phase, intrinsic genetic programs give rise to developmentally induced astrocyte (DIA) subsets, which form the framework for later astrocyte functional specialization. Subsequently, these DIA populations can be shaped by environmental stimuli, including neurotransmitters, cytokines, or metabolic factors, resulting in the occurrence of specific stimulus-induced astrocyte (SIA) subtypes [270,271,272,273]. The developmental origins of astrocyte heterogeneity are supported by the region-specific expression of genes such as Paired box 6 (Pax6), NK6 Homeobox 1 (Nkx6.1), Reelin, Slit Guidance Ligand 1 (Slit1), and Semaphorin 3A (Sema3a) in spinal cord astrocytes [274,275,276]. These findings suggest that an aberrant neurodevelopmental process may lead to impaired DIA maturation in specific brain regions (e.g., the PFC or hippocampus). This, in turn, may render these regions more susceptible to environmental influences, ultimately contributing to structural and functional abnormalities in the brain which are implicated in the schizophrenia pathophysiology.

Given the differences in gene expression patterns across the lifespan, as well as the sex-dependent variations in clinical presentation, age of onset, and responses to antipsychotic treatments in schizophrenia [277,278], a recent study has identified sexual dimorphism in the expression of genes related to neurodevelopment in iPSC-derived astrocytes [234]. Understanding these properties may provide valuable insights into how astrocytes shape the brain’s development in schizophrenia [279,280], contributing to sex differences in its clinical manifestation.

Postmortem studies in individuals affected by schizophrenia have revealed aberrant neurodevelopmental gene expression. Some studies conducted in vitro with iPSCs derived from schizophrenia patients confirmed the differentially expressed genes related to glial differentiation and metalloproteinase activity between astrocytes developed from schizophrenia patients and those derived from healthy controls [62,281]. After that, the engraftment of human GPCs-iPSCs from schizophrenia patients into wild-type mice induced a range of behavioral abnormality-like anxious behavior, social deficits, and impaired prepulse inhibition (PPI), which are among the key features of the disease [282]. An RNA sequencing study demonstrated that GPCs derived from schizophrenia patients exhibited the downregulation of genes associated with astroglia differentiation. These findings could suggest that impaired astrocyte differentiation may be an early event in schizophrenia, followed by the astrogliosis observed in these patients, which could be a secondary event occurring later in the disease’s progression [3,62,279,283,284,285,286,287,288,289]. Furthermore, astrocytes are also involved in regulating neuronal circuit formation by controlling D-serine availability, which preferentially promotes the diffusion of NR2B over NR2A NMDAR subunits [290,291], considering that NR2B is more highly expressed during early neurodevelopment in the hippocampus, whereas NR2A expression increases at later stages [292,293].

In conclusion, the astrocytes’ aberrant maturation and the wrong timing of this process trajectory could significantly impact synaptogenesis and neuronal circuit formation, and underlines the critical role that astrocytes play in guiding neural connectivity and synaptic development [120,294,295,296,297,298].

### 3.6. Astrocyte-Related Intracellular Mechanisms as Putative Pharmacological Targets

Antipsychotics remain the cornerstone of pharmacological treatment for schizophrenia. Although these drugs primarily act upon neuronal membrane receptors (mainly D2Rs) and intracellular pathways (e.g., cAMP-PKA, Beta2arrestin, and PLC regulation), glial cells, including astrocytes, have emerged as potential targets to enhance treatment efficacy [299]. In fact, beyond their direct effects on neurons and indirect impact on glial cells, as conceptualized in the tripartite synapses, growing evidence suggests that antipsychotics may also exert direct actions on astrocytes [299]. This effect may occur through the direct interaction with the D2R via cyclic adenosine monophosphate (cAMP), which is abundantly expressed on astrocytes, or by modifying traditional neuron–glia communication [300,301,302,303].

**Figure 2:**
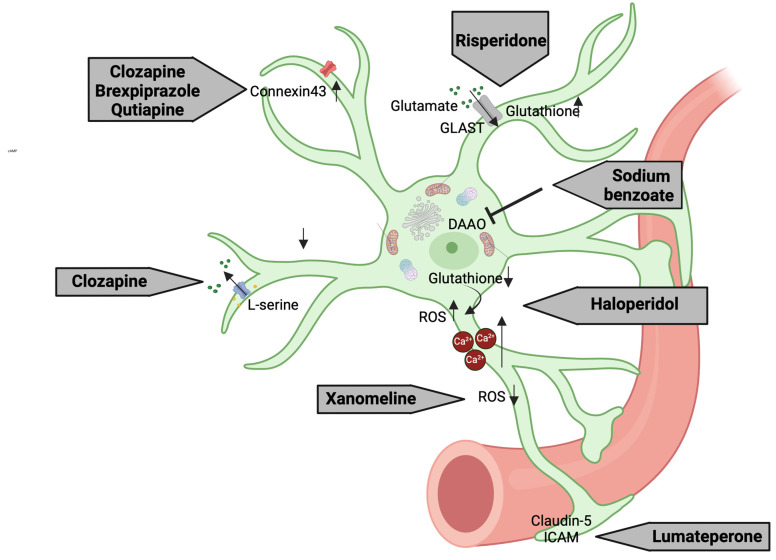
Antipsychotics’ effects on astrocytic cells. NFκB, nuclear factor kappa B; Akt, protein kinase B; ERK, extracellular signal-regulated kinase; GLAST, glutamate transporters; DAAO, D-amino acid oxidase; ROS, reactive oxygen species. Created with Biorender.com.

The dopamine antagonism exerted by antipsychotics can increase glutamate levels, potentially leading to neurotoxicity [183,304]. In contrast, astrocytes support neuronal function and prevent glutamate excitotoxicity by converting glutamate into glutamine [305]. Clozapine, along with quetiapine and brexpiprazole, have been shown to enhance tripartite synaptic glutamatergic transmission through the activation of Connexin43 (Cx43) hemichannels [306] (Figure 2 and Table 3). It has been found to also increase the release of L-serine from astrocytes and help to restore glutamate and serine levels in astrocytes derived from the iPSCs of schizophrenia patients [307]. Risperidone has been shown to both increase glutamate uptake by the C6 astroglial cells [306] and reduce oxidative stress through increasing glutathione (GSH) levels compared to haloperidol [308,309,310,311,312], effects that potentially contribute to the pathogenesis of extrapyramidal side effects [313] (Figure 2 and Table 3). Additionally, haloperidol was able to induce cytotoxicity through increasing intracellular Ca^2+^ levels, findings which were also reported in the GBM 8401 human glioblastoma cell line after chlorpromazine administration (Table 3) [310,311].

On the other hand, sodium benzoate—an add-on treatment for treating schizophrenia—acts as an inhibitor of D-amino acid oxidase (DAAO), a peroxisomal flavoenzyme that is almost exclusively expressed within astrocytes, therefore increasing D-amino acid levels (Figure 2 and Table 3) [314]. Lumateperone helps to maintain BBB integrity by regulating proteins like claudin-5 and intercellular adhesion molecule 1 (ICAM-1), indirectly supporting astrocytic health [315] (Figure 2 and Table 3). Considering that xanomeline has proved effective in preventing cortical cell apoptosis and ROS production in vivo and in vitro [316], and taking into account the neuroprotective role exerted by astrocytes, it is reasonable to hypothesize an astrocyte-mediated effect in the antipsychotic’s properties.

Taken together, growing evidence highlights the role of astrocytes in mediating antipsychotic effects, particularly through modulation of glutamate uptake, D-serine release, and redox mechanisms. These glial cells are complementary to neuronal activity, supporting structural and metabolic functions, while also playing central roles in chemical regulation and neuroprotection.

**Table 3:** Effects of antipsychotic drugs on astrocytic functions. Cx43, connexin 43; GLT-1, glutamate transporter 1 (EAAT2); D-serine, D-2-amino-3-hydroxypropanoic acid (NMDA receptor co-agonist); GLAST, glutamate aspartate transporter (EAAT1); GSH, glutathione; Ca^2^⁺, calcium ion; DAAO, D-amino acid oxidase; claudin-5, tight junction protein claudin-5; ICAM-1, intercellular adhesion molecule 1; ROS, reactive oxygen species; BBB, blood–brain barrier, hiPSC, human-induced pluripotent stem cell.

Drug	Doses	Duration of Treatment	Preclinical Setting	Astrocytes’ Effects	Molecular Effect	References
Clozapine	30 μM	48 h	Primary astrocyte cultures from the cerebral cortex of 1-day-old Sprague Dawley rats	Astrocyte activation and glutamate modulation	Reduction in GLT-1 levels of about 50%; no changes in GLAST levels	[317,318]
	unknown	unknown	hiPSC-derived astrocytes from schizophrenia patients		Reduction in L-serine levels	[307]
	3 μM	7 days	Primary astrocyte cultures		Increased Cx43 expression	[306]
Brexpiprazole	0.3 μM			Astrocytic glutamate modulation		
Quetiapine	1 μM			Astrocytic glutamate release		
Risperidone	0.1, 1, and 10 μM	acute	C6 glial cell line	Glutamate uptake and antioxidant defense	Increased GSH levels	[305]
Haloperidol	10 μM				Increased ROS levels	
	20–40 μM	acute	Gibco^®^ human astrocytes	Astrocyte toxicity and glutamate transport	Increased Ca^2+^ levels	[311]
Lumateperone	3 mg/kg	acute	LPS-injected C57BL/6 mice	BBB integrity	Modulation of claudin-5 and intercellular adhesion molecule 1	[315]
Xanomeline	300 nM	10 min	Prefrontal brain slices of mice	Neuroprotection	Reduction in ROS production and inhibition of astrocyte-mediated apoptosis	[316]

## 4. Discussion

Although the neurodevelopmental and the neurodegenerative hypothesis of schizophrenia may appear opposed, it is conceivable that both may sustain aspects of a common pathology in a different stage of the disease’s trajectory wherein early aberrant neurodevelopment may lay the groundwork for later neurodegenerative changes [3,6]. Astrocytes may emerge, among other glial cells and ECM elements, as the structural and functional link between these two models and could be a potential future therapeutic way of targeting the glial component of the tripartite synapse.

The role of astrocytes in the pathophysiology of schizophrenia is sustained by alterations in their density, morphology, and specific marker expression across the brain regions discussed as being involved in the schizophrenia pathophysiology (e.g., ACC, PFC, hippocampus) [42,47,48,319]. These changes may lead to astrocytic dysfunction in regulating neurotransmitter systems, particularly glutamate, GABA, and dopamine [102,115,116,117]. We then focus on the molecular mechanisms underlying the structural and functional alteration of astrocytes that emerge during neurodevelopment (e.g., BDNF-TrkB pathway, TGF-β-TBR1/2 pathway, NLG) [88,89,91,92] (Table 2). Unlike neurons that are generally postmitotic, astrocytes retain their proliferative capacity at birth and maintain a more plastic methylome, which remains labile even in adulthood [320]. This capability is related, among other factors, to a different switch of the glial line driven by nuclear factor I-A (NFIA) and transcription factor Sox 9, Notch signaling, and transcriptional repressor N-CoR that converge to the Janus kinase (JAK)/signal transducer and activator of transcription (STAT) (JAK/STAT) signaling cascade [321,322,323]. Disruptions in these key regulatory pathways may result in impaired astrocyte differentiation, abnormal glial–neuronal interactions, and altered neuroinflammatory responses. Astrocytic dysfunctions play a role in the pathophysiology of schizophrenia, potentially serving as an initial contributor to DIA and SIA subtype differentiation, while environmental factors, including exposure to neurotoxic agents, serve as secondary influences that worsen these dysfunctions [64,96,102,115,116,117,162,270]. Collectively, these factors may increase the susceptibility of brain cells to degenerative processes, suggesting that although schizophrenia is not traditionally classified as a neurodegenerative disorder, it may involve unconventional and non-canonical neurodegenerative mechanisms [11]. Nonetheless, it remains unclear whether astrocytic alterations are primary drivers, or represent the cumulative, long-term consequences of the disorder. Aberrant gene expression and impaired astrocyte differentiation, observed in the postmortem brains of schizophrenia patients, point to early neurodevelopmental disruptions that may predispose individuals to later structural and functional abnormalities, including those coincident with astrogliosis [62,279,283,284,285,286,287,288,289]. These findings raise the possibility that astrocytic-related processes could be therapeutically targeted by antipsychotic treatments currently used in schizophrenia and TRS. This perspective shifts the paradigm beyond the classical view of antipsychotic efficacy as being solely dependent on neuronal targets, toward a broader understanding that includes the direct modulation of astrocyte function. Indeed, increasing evidence suggests that antipsychotics can directly influence astrocyte-mediated processes such as glutamate uptake and D-serine release, opening new avenues for improving treatment efficacy [306,307,317,324].

In vitro studies indicate that glial progenitors in cellular culture derived from patients affected by schizophrenia exhibit the reduced expression of genes involved in astrocyte differentiation and altered metalloproteinase activity [62,281]. These findings may indicate an early defect in astrocyte maturation in this clinical population, leading to impaired synaptogenesis and neural network formation, as demonstrated by preclinical studies reporting behavioral abnormalities such as social deficits and impaired PPI in a rodent model [282]. Additionally, aberrant metalloproteinase production has been implicated in the progression of neurodegenerative diseases, such as Alzheimer’s disease [281], likely through mechanisms involving neuroinflammation, the disruption of the BBB, and synapse degradation [221]. These findings align with the idea that alterations during the early developmental stages of astrocytic progenitors (such as the impaired expression of D-serine synthesis and the downregulation of glutamate transporters, GLAST) may contribute to non-canonical degenerative processes, thereby exacerbating not only the occurrence of early-stage symptoms due to aberrant pruning processes but also the symptomatology characteristic of the chronic stages of schizophrenia. Studies should be undertaken to clarify if and to what extent D-amino acids, whose role in schizophrenia pathophysiology and treatment, especially of TRS forms, has sparked interest in recent decades, may affect astrocyte function.

The underpinning molecular mechanisms could be related, among others, to PSD function at the tripartite synapses, because of its role in astrocyte–neuron communication that is impaired in schizophrenia and particularly in TRS conditions, considering the effect of clozapine in modulating astrocytic hemichannel activity, and enhancing glutamate and D-serine release [307]. These two molecules are strongly deregulated in schizophrenia for the aberrant buffering effect exerted by dysfunctional astrocytes in both the glutamate and D-serine levels responsible for aberrant NMDAR-dependent synaptic plasticity and cognitive processes in humans, which are related to the excitotoxicity and neuronal damage reported in neurodegenerative diseases such as amyotrophic lateral sclerosis and Alzheimer’s disease [325,326]. On the other hand, hormones impact astrocytic development, resulting in sex-specific patterns of synaptic regulation, which influence phenotypic presentation differences between sex, age of onset, and prognosis [327]. However, these molecules are also responsible for the difference in vulnerability to neurodegenerative processes, considering the major incidence of Alzheimer’s after menopause, becoming a putative convergence molecular factor between the neurodevelopmental and neurodegenerative processes that underlie the schizophrenia clinical presentation spectrum [328,329].

Considering the reduced expression of GLAST and altered D-serine release associated with astrocytic dysfunction in schizophrenia, the final question we aim to address concerns the potential role of astrocytes in pharmacological treatment, particularly in the context of precision psychiatry for schizophrenia and TRS. Evidence indicates that antipsychotics can modulate astrocytic functions [306,317,324]. Clozapine can reduce GLT-1 levels from the baseline (around 50%), with no changes in GLAST levels, and can reduce glutamate uptake in a dose-dependent manner in primary astrocyte cultures from the cerebral cortex [317]. Additionally, in the same preclinical setting, chronic clozapine downregulated GLT-1, synaptosome-associated protein of 25 kDa (SNAP-25), and vesicular glutamate transporter 1 (VGLUT1), along with increasing synaptophysin [324]. In contrast, acute and subchronic clozapine administration enhanced L-glutamate release through the activation of Cx43 hemichannels. While quetiapine and brexpiprazole had no acute effects, subchronic treatment enhanced astroglia L-glutamate release through Cx43 activation in the primary astrocyte culture [306]. Chronic haloperidol has been shown to negatively impact the neuronal environment inducing cytotoxicity via the deregulation of astrocytes’ glutamate levels because of a reduction in GLT-1 activity [330,331].

These findings suggest that novel treatment approaches could complement current pharmacological treatments by directly targeting astrocytic functions. Potential strategies may include enhancing glutamate clearance and buffering by targeting astrocytic glutamate and excitatory amino acid transporters (including EAAT1 and EAAT2); modulating the release of neuromodulators, such as D-serine; reducing astrocyte-mediated neuroinflammation and oxidative stress; and supporting BBB integrity through the modulation of metalloproteinase and junctional protein stability [103,315]. This effect is related to the activation of astrocytes’ Shh pathway: astrocytes release Shh, which binds the endothelial Patched-1 causing the release of Smoothened which is responsible for the increase in junctional protein expression (e.g., ICAM-1, claudin-3, and GLUT-1) and the downregulation of cytokines, avoiding neuroinflammation processes via the activation of the transcription factor zinc finger protein GLI1 (GLI-1) [105].

This work has several limitations that need to be considered. First, a large part of the data we reviewed here is based on the modeling of preclinical studies, with all the difficulties of translating the findings to humans. Additionally, we focused on the role of astrocytes, and explored little of the involvement of other glial cells, such as microglia and oligodendrocytes, which also likely contribute to the schizophrenia pathophysiology. Furthermore, our analysis centered on two key structures relevant to schizophrenia—PSD and tripartite synapses—in the much larger framework of schizophrenia’s synaptic pathology. Lastly, while this article critically examined the current understanding of a potential molecular continuum from early neurodevelopment to late-stage non-canonical neurodegeneration, the absence of longitudinal studies investigating astrocyte dysfunction across this trajectory limited our possibility of inferring long-term experimental paradigms whose implementation is warranted.

## 5. Conclusions

Multiple lines of evidence support the viewpoint of there being a possible trajectory in schizophrenia etiopathogenesis, primed in the early stage by the brain’s abnormal development setting a dysfunctional environment that, in turn, results in aberrant neuronal circuits and early aging as well as the non-canonical neurodegenerative process. In this framework, astrocytes, together with other glial cells, could represent a point of convergence between the two different stages of the disease, contributing at an early stage to aberrant neurodevelopment and favoring late non-canonical mechanisms of accelerated aging and neurodegeneration. Finally, it is conceivable that potential novel astrocyte-targeted strategies should be explored, especially for those forms of schizophrenia that are not responsive to preset pharmacological agents.
